# Transcriptome Analysis of Hypertrophic Heart Tissues from Murine Transverse Aortic Constriction and Human Aortic Stenosis Reveals Key Genes and Transcription Factors Involved in Cardiac Remodeling Induced by Mechanical Stress

**DOI:** 10.1155/2019/5058313

**Published:** 2019-10-27

**Authors:** Peng Yu, Baoli Zhang, Ming Liu, Ying Yu, Ji Zhao, Chunyu Zhang, Yana Li, Lei Zhang, Xue Yang, Hong Jiang, Yunzeng Zou, Junbo Ge

**Affiliations:** ^1^Department of Endocrinology and Metabolism, Fudan Institute of Metabolic Diseases, Zhongshan Hospital, Fudan University, Shanghai, China; ^2^Shanghai Institute of Cardiovascular Diseases, Shanghai Clinical Bioinformatics Research Institute, Zhongshan Hospital, Shanghai Medical College of Fudan University, Shanghai, China; ^3^Department of General Practice, Zhongshan Hospital, Shanghai Medical College of Fudan University, Shanghai, China

## Abstract

**Background:**

Mechanical stress-induced cardiac remodeling that results in heart failure is characterized by transcriptional reprogramming of gene expression. However, a systematic study of genomic changes involved in this process has not been performed to date. To investigate the genomic changes and underlying mechanism of cardiac remodeling, we collected and analyzed DNA microarray data for murine transverse aortic constriction (TAC) and human aortic stenosis (AS) from the Gene Expression Omnibus database and the European Bioinformatics Institute.

**Methods and Results:**

The differential expression genes (DEGs) across the datasets were merged. The Venn diagrams showed that the number of intersections for early and late cardiac remodeling was 74 and 16, respectively. Gene ontology and protein–protein interaction network analysis showed that metabolic changes, cell differentiation and growth, cell cycling, and collagen fibril organization accounted for a great portion of the DEGs in the TAC model, while in AS patients' immune system signaling and cytokine signaling displayed the most significant changes. The intersections between the TAC model and AS patients were few. Nevertheless, the DEGs of the two species shared some common regulatory transcription factors (TFs), including SP1, CEBPB, PPARG, and NFKB1, when the heart was challenged by applied mechanical stress.

**Conclusions:**

This study unravels the complex transcriptome profiles of the heart tissues and highlighting the candidate genes involved in cardiac remodeling induced by mechanical stress may usher in a new era of precision diagnostics and treatment in patients with cardiac remodeling.

## 1. Introduction

Heart failure, the end stage for most cardiac diseases, is a clinical syndrome in which the heart is unable to provide sufficient blood flow to meet physiologic requirements of the body. Prior to clinical symptoms or signs of heart failure, the body tries to maintain adequate tissue perfusion using several mechanisms, including the Frank–Starling mechanism and neurohormonal activation, which lead to cardiac remodeling [1].

Cardiac remodeling is a process in which genomic changes occur. Physiologically, signaling and transcriptional control involve precise programs of gene activation and suppression [2]. Transcriptional changes in response to pathological stress might promote deterioration of cardiac remodeling. It has been shown that preventing the genomic changes may be a promising therapeutic approach [2–4].

The murine transverse aortic constriction (TAC) is a commonly used experimental model for mechanical stress-induced cardiac remodeling, which clinically mimics the aortic stenosis (AS). TAC initially leads to compensated hypertrophy of the heart and is often associated with a temporary enhancement of cardiac contractility. In the end stage, the response to chronic hemodynamic overload becomes maladaptive, leading to cardiac dilatation and heart failure. The murine TAC model has since been extensively used as a valuable tool to mimic human cardiovascular diseases and elucidate fundamental signaling processes involved in the cardiac hypertrophic response and heart failure development. It provides a more reproducible model of cardiac hypertrophy and a more gradual time course for the development of heart failure [5].

DNA microarrays facilitate measurement of the expression levels of large numbers of genes simultaneously. Recent data underscored the significance of genomic mechanisms in regulating gene expression programs in cardiac pathology [2]. A number of studies have investigated the genomic changes of the heart in the process of cardiac remodeling.

To investigate the genomic changes in the process of cardiac remodeling induced by mechanical stress systematically and without bias, we collected and analyzed DNA microarray data for cardiac remodeling induced by TAC and AS from the Gene Expression Omnibus (GEO) database and European Bioinformatics Institute (EBI). As a result, we found a set of gene expression changes in the cardiac pathologic remodeling induced by mechanical stress that shared some common transcription factors (TFs) with each other.

## 2. Methods

### 2.1. Microarray Data Collection and Preprocessing

The gene expression profiles were screened and downloaded from the National Center for Biotechnology Information GEO database and the EMBL-EBI. To explore cardiac remodeling under mechanical stress, the murine TAC datasets and the human AS datasets were included. The TAC datasets in which hypertrophic genes NPPA, NPPB, ACTA1, and MYH7/MYH6 remained unchanged were excluded from analysis. Datasets with the number of samples in each group of <3 were also excluded.

### 2.2. DEG Analysis

GEO series were analyzed separately using the online GEO2R tool with default parameters (https://www.ncbi.nlm.nih.gov/geo/geo2r/), in which the empirical Bayes algorithm (function “eBayes”) in the limma package was used to detect differentially expressed genes between the TAC model or AS patients and controls. In the murine model analysis, the genes with a *P* value (Bayes test) of <0.05 were considered as DEGs for the multiple intersection of different datasets.

Since datasets were from different research centers, group variation was present. It was not possible to conduct the data analysis on interdatasets. Considering these limitations, we obtained only the average values of logFC from each dataset to represent the expression levels [6]. In the analysis of AS patients, significantly changed genes were defined by a logarithmic-transformed fold-change absolute value (log2(FC)) ≥ 1 and a *P* value of ≤0.05.

### 2.3. Venn Analysis

Comparative analysis was carried out with the InteractiVenn tool (http://www.interactivenn.net/) [7] and Bioinformatics and Evolutionary Genomics tool (http://bioinformatics.psb.ugent.be/webtools/Venn/).

### 2.4. GO Analysis

DAVID was employed to perform the GO analysis for biological processes and pathway enrichment. To plot the BPs of the DEGs involved, we used the clusterProfiler package [8].

### 2.5. PPI Network Construction Analysis

STRING online tool (string-db.org) [9] was used to establish a PPI network for the murine TAC model. Cytoscape software [10] was used to establish a PPI network for DEGs of AS patients, with the cutoff of a combined score of >0.4. The network analyzer plug-in for the Cytoscape software was used to analyze the topological property of the networks [6]. Genes with the edge degree of ≥7 were defined as hub genes in this article.

### 2.6. TF Analysis

The Transcriptional Regulatory Relationships Unraveled by Sentence-based Text mining version 2 database (https://www.grnpedia.org/trrust/) [11] was used to predict regulation of TFs based on the lists of upregulated and downregulated genes generated across the microarray datasets. Significant TFs and potentially regulated genes were identified based on a multiple parameters, *P* < 0.05 [12]. We used the “igraph” package in R to visualize the output results.

## 3. Results

### 3.1. Datasets Involved in This Study

We searched a total of 14 datasets, which included a model of murine cardiac remodeling induced by TAC and utilized a microarray to detect differential expression genes (DEGs) in an unbiased manner. Four datasets for the early cardiac remodeling and seven datasets for the late cardiac remodeling were used (Table 1).

### 3.2. Genomic Changes in the Early Hypertrophic Response Stage

The period within two weeks after the TAC operation was defined as the early stage of cardiac remodeling, characterized by compensated hypertrophic remodeling. Four datasets included the microarray data from analysis of this period.

**Figure 1:**
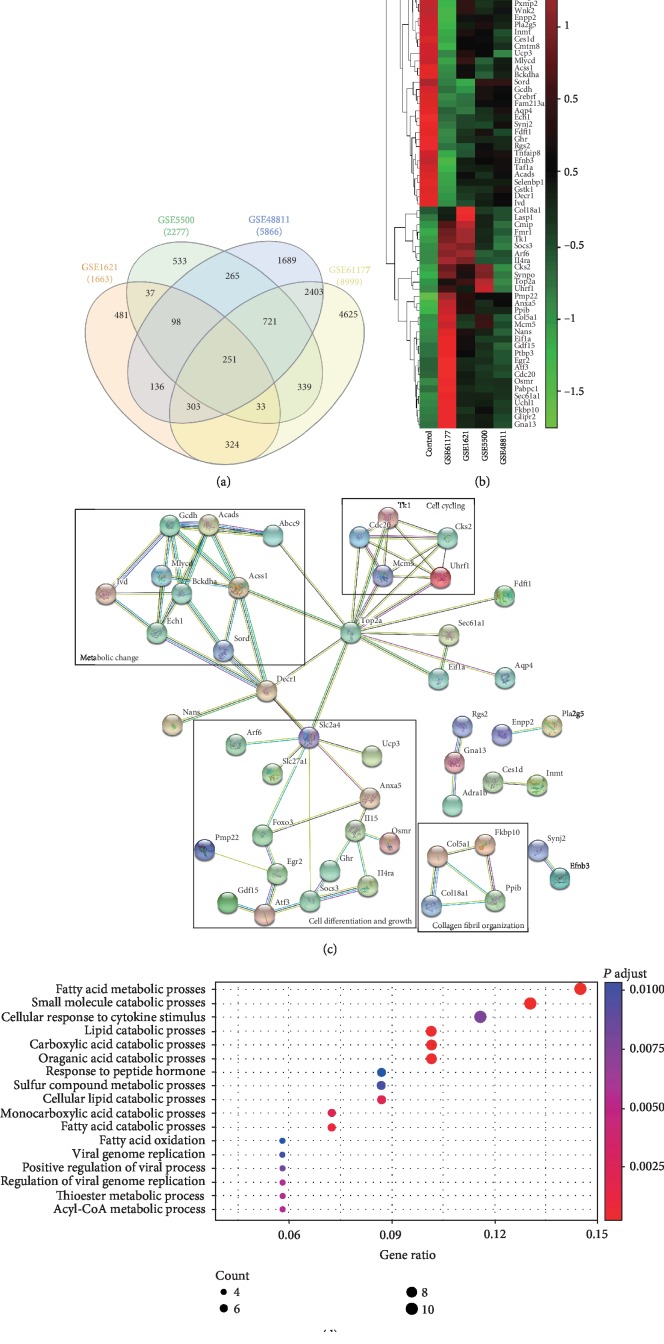
Shared DEGs in the four datasets for the early stage of cardiac remodeling. (a) Venn diagram showed 251 shared DEGs. (b) Heatmap for 74 DEGs with same trends from the four datasets. (c) Network diagram of 74 DEGs with same trends in the early stage of cardiac remodeling. (d) Plotted biological processes for 74 DEGs.DEG: differential expression gene.

The four datasets shared 251 significant DEGs (Figure 1(a)), among which only 74 exhibited similar trends. The heatmap showed DEGs with the same trends across the four datasets (Figure 1(b)). The 74 DEGs were analyzed using the STRING online tools (Figure 1(c)). To show the main biological processes involving DEGs, we performed Gene Ontology (GO) analysis using the Database for Annotation, Visualization and Integrated Discovery (DAVID) ([Supplementary-material supplementary-material-1]), with the results represented in Figure 1(d).

Intersections among the four datasets comprised only a small portion of each dataset. However, among the intersections, the number of DEGs with same trends was even smaller. STRING analysis showed that the 74 DEGs were mainly concentrated in metabolic changes, cell differentiation and growth, cell cycling, and collagen fibril organization (Figure 1(c)). The BP enrichment of the DEGs mainly occurred during the lipid metabolic change ([Supplementary-material supplementary-material-1]).

### 3.3. Genomic Changes in the Late Stage of Cardiac Remodeling

**Figure 2:**
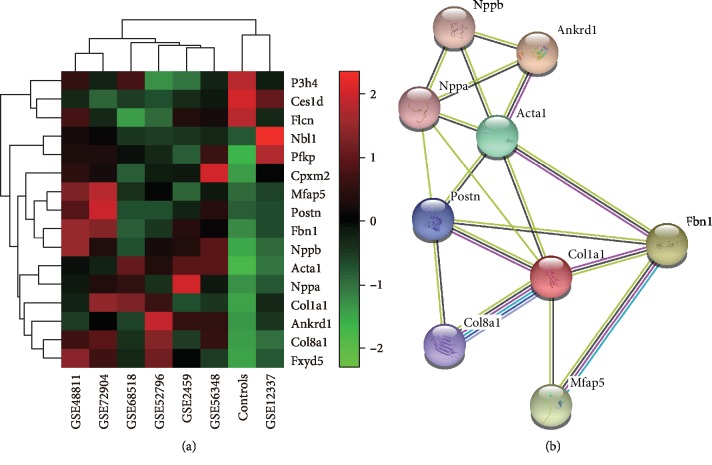
DEGs with same trends in the late stage of cardiac remodeling. (a) Heatmap for 16 shared DEGs from the four datasets. (b) Network diagram for DEGs in the late stage of cardiac remodeling.

We then analyzed the datasets detecting DEGs more than four weeks post-TAC, which represented gene changes in the late stage of cardiac remodeling. The analysis involved seven datasets. The Venn analysis of DEGs is shown in [Supplementary-material supplementary-material-1]. In the seven datasets, only 16 DEGs exhibited the same trends, which is shown as a heatmap in Figure 2(a). We consequently performed the protein–protein interaction (PPI) analysis of the 16 DEGs using the STRING tools (Figure 2(b)). Biological processes involving these 16 DEGs were concentrated mainly in collagen biosynthesis and hypertrophic marker molecules, such as NPPA, NPPB, and ACTA1 (Table 2).

Although intersection of the seven datasets credits the genes involved in the TAC-induced cardiac remodeling, its comprehensiveness may be attenuated for the multiple intersections. Additionally, we performed PPI and BP analyses for the DEGs in the intersection of at least six sets ([Supplementary-material supplementary-material-1] and [Supplementary-material supplementary-material-1]). The results showed collagen biosynthesis process, innate immune response, metabolic changes, and ion transmembrane transport to be the main changes involved in the late remodeling stage.

### 3.4. Microarray Data Analysis of the Human Heart Tissue from AS Patients

TAC is a common model used to investigate cardiac remodeling and heart failure. Clinically, heart failure is a syndrome with multiple heterogeneous etiologies. Hypertension and AS are the main heart failure types induced by mechanical stress, a model of TAC.

**Figure 3:**
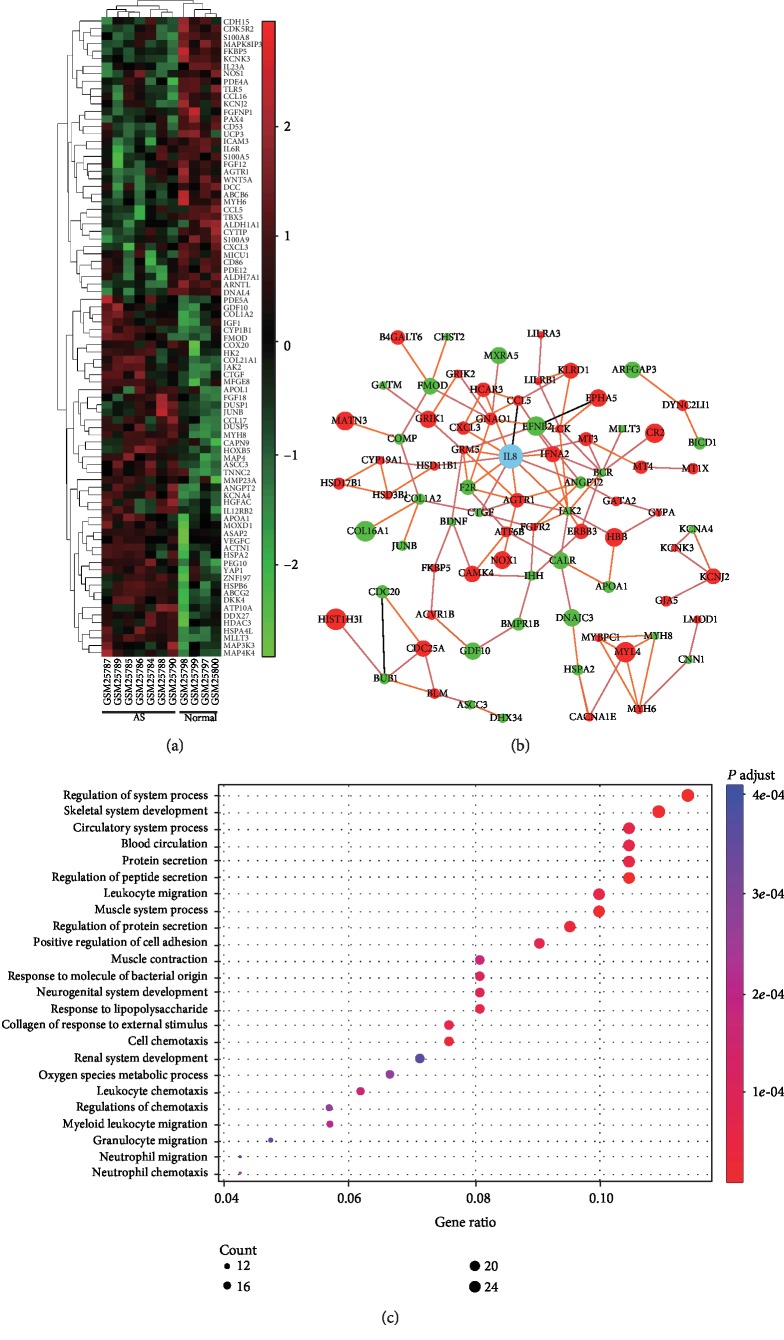
DEGs in the human heart tissues of AS patients. (a) Heatmap for heart tissue DEGs from AS patients. (b) Network diagram for DEGs in AS patients. (c) Plotted BP for DEGs. AS: aortic stenosis; DEG: differential expression gene; BP: biological process.

To investigate the DEGs involved in human heart failure induced by mechanical stress, we analyzed GSE1145, in which datasets for the heart tissues from AS patients were utilized. The total DEG count was 252. Some of these genes are represented by a heatmap in Figure 3(a). BP analysis showed that the genes mainly enriched the inflammatory process, in addition to playing a role in muscular hypertrophic changes (Figure 3(c)). In the PPI analysis, four genes were identified as hub genes with the edge degree ≥ 7. According to the edge degree rank, the four hub genes were IL-8, JAK2, AGTR1, and BCR. IL-8, in particular, might play an important role in the development of mechanical stress induced by AS. However, these four genes were not involved in the analysis of ischemic cardiomyopathy [6], implying a distinct pathogenesis between these two cardiomyopathies.

Compared with the mechanical stress-induced cardiac remodeling in mice, few DEGs or BPs overlapped between the murine TAC model and AS patients, in which the effects of clinical medication had to be excluded.

### 3.5. TF Analysis

**Figure 4:**
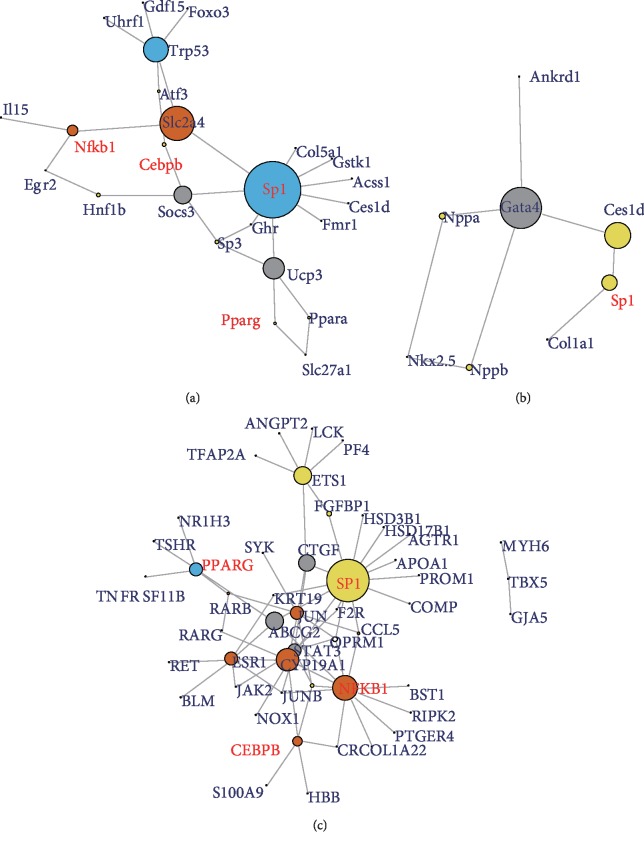
Prediction of the TFs of DEGs in mice and human hypertrophied heart induced by mechanical stress. (a) TFs involved in the early cardiac remodeling of mice. (b) TFs involved in the late cardiac remodeling of mice. (c) TFs involved in the hypertrophied patients. The red character showed the mutual TFs in the mice and human. DEG: differential expression gene; TF: transcription factor.

We also predicted the TFs regulating DEGs using the data from the TAC model and AS patients. Although the DEGs shared little overlap between human and murine mechanical stress-induced hypertrophic heart tissue, there were four TFs (SP1, CEBPB, PPARG, and NFKB1) in common between early cardiac remodeling (Figure 4(a)) and AS patients (Figure 4(c)). The TFs predicted in the late cardiac remodeling were few for a little set of DEGs (Figure 4(b)).

The most prominent TF was SP1, which is involved in many cellular processes, including cell differentiation, cell growth, apoptosis, immune response, response to DNA damage, and chromatin remodeling. Activity of CEPPB and NFKB1 is important in the regulation of genes involved in immune and inflammatory responses. PPARG is a regulator of metabolic changes.

## 4. Discussion

Using the data from the high throughput DNA microarray analysis, we were able to systemically reveal genomic changes in a disease, so that potential therapeutic targets could be identified in the future.

In this study, we investigated the datasets for TAC, a typical model to explore cardiac remodeling. We divided the datasets into early and late phases of cardiac remodeling, according to data from mice that succumbed days after the TAC operation. After analysis of the data, we found common gene changes within different datasets, which mainly converged on matrix remodeling, metabolic changes, and mechanical response.

Genomic changes in cardiac remodeling have recently gained attention from researchers and their modulation has been widely investigated. The methylation of DNA [23] and histones [24], acetylation of chromatin and facilitation of transcriptional activation [3, 25], and chromatin structural remodeling [26] all result in genomic changes and lead to heart failure. Suppression of genomic changes could ameliorate cardiac remodeling. Thus, it is important to determine the genomic changes taking place during heart failure. Our study represents the first attempt to systematically elucidate these changes.

In a murine model, the genome is altered in the early stages of metabolic changes, cell differentiation and growth, cell cycling, and collagen fibril organization. A recent study has revealed that cyclins and TGF-*β* that have terminally exited the cell cycle can unlock the proliferative potential in the myocardium. Moreover, their overexpression could improve the cardiac function [27]. The PPI analysis showed that SLC2A4 and TOP2A are the two centers of genomic change. SLC2A4, also known as GLUT4, is a glucose transporter that facilitates the metabolic switch to glucose in cardiac remodeling. TOP2A (DNA topoisomerase II-alpha) controlled the topological states of DNA by transient breakage and subsequent rejoining of DNA strands that facilitated cellular mitosis, chromatin remodeling, and gene transcription [28–30].

There were fewer gene changes in the late stages of cardiac remodeling, where only 16 genes exhibited similar trends in all of the datasets. The upregulated genes ACTA1, NPPA, NPPB, POSTN, COLIA1, and COL8A1 were regarded as molecular markers in the pathologic process of cardiac remodeling. Ces1d was downregulated in all datasets. It has been shown to be involved in lipolysis, the process whereby the adipocyte hydrolyzes stored triglycerides into fatty acids to be used as fuel in times of need [31, 32], in correlation with the opinion that the switch from fat to glucose is an approach that could be taken to improve cardiac remodeling [33]. FLCN, the inactivation of which could potentially lead to cardiac remodeling [34], was also downregulated. Research reports involving other genes from the set of 16 identified in this study, including P3H4, ANKRD1, CPXM2, FBN1, FXYD5, MFAP5, NBL1, PFKP, and SLMAP, were rare. These genes are therefore worth exploring further.

To delineate the correlation between the murine model and clinical patients, we analyzed datasets from AS patients mimicked by TAC [5]. The results showed that the genomic changes in AS biological processes were mainly in inflammation. Results from the PPI analysis identified IL-8, JAK2, AGTR1, and BCR to be the centers of genomic changes, in which AGTR1 was the target of hypertension, the common cause of cardiac remodeling triggered by mechanical stress. Sartans, antagonists of AGTR1, are the cornerstone of medication for hypertension. Accordingly, IL-8, JAK2, and BCR might be therapeutic targets for hypertension, which were not detected in the microarray data from the TAC model.

The DEG intersections between the murine TAC model and AS patients were few. However, predicted TFs from DEGs SP1, CEBPB, PPARG, and NFKB1 were the common TFs between the two species. Unsurprisingly, they were either regulators in metabolic changes or pivotal hubs in inflammatory response. SP1 has been reported to contribute to the regulation of critical molecules involved in cardiac remodeling [35, 36]. The mice downregulated of CEBPB has been reported to display substantial resistance to cardiac failure upon pressure overload, indicating its repression of cardiomyocyte growth and proliferation in the adult mammalian heart [37]. The PPAR gene pathway coordinately act to regulate cellular processes central to glucose and lipid metabolism [38]. NFKB1 signaling also is critical for both cardiac remodeling and hypertrophy [39].

## 5. Conclusion

In conclusion, we offer a novel and comprehensive analysis of gene expression profiles using microarray DNA datasets in cardiac remodeling induced by mechanical stress, mimicking hypertension. Genes involved in metabolic changes, extracellular matrix remodeling, and cell differentiation and growth were significantly changed in the heart tissue from the murine TAC model. Compared to results from the TAC model, the significantly changed genes in patients suffering from AS were mostly enriched during the inflammatory biological processes. The analysis will provide valuable information for future research on the molecular mechanisms of cardiac remodeling and offer clues for the discovery of novel therapeutic strategies.

## 6. Limitations

Although our analysis was comprehensive, with high throughput and a large sample size, some limitations were still present.

Classification of the cardiac remodeling stages in the murine TAC model was performed on the basis of time passed postoperation. No echo or histological standard was used. The TAC model was performed using a standard operating procedure [5]. The period within two weeks after the operation was regarded as hypertrophic or compensatory stage, while the period past four weeks was considered to be the dilated or decompensated stage.

Furthermore, the data for patients suffering from AS were extracted from GSE1145, which lacks detailed clinical information as no research was published using this dataset. However, the DEGs mainly involved in the inflammatory biological processes were similar to a previous study of cardiac remodeling [6, 40].

Despite these limitations, the comprehensive analysis of microarray data in this study makes the results compelling.

## Figures and Tables

**Table 1:** Studies that included murine model of cardiac remodeling induced by TAC using DNA microarray.

Accession number	Stain	Days post TAC	Sample volume	Citation	Hypertrophic gene expression	
GSE61177	C57BL/6	3d	4 vs 3	[13]	Elevated	Enrolled
GSE1621	FVB	10d	4 vs 4	[14]	Elevated	Enrolled
GSE5500	C57Bl6/J–FVB/N	7d	4 vs 6	[15]	Elevated	Enrolled
GSE415	C57BL/6	7d	4 vs 4	[16]	Unchanged	Excluded
GSE5129	C57BL/6	7d	1 vs 1	[17]		Excluded for small sample size
GSE48110	C57Bl/6	3d, 11d, &28d	3 vs 3 for each time point	[3]	Elevated	Enrolled
GSE38733	Not shown	28d	1 vs 1	Unpublished		Exclued for small sample size
E-MTAB-2732	C57BL/6	Ambiguous	Ambiguous	Unpublished		Exclueded
GSE12337	C57BL/6	28d	4 vs 4	[18]	Elevated	Enrolled
GSE2459	FVB	30d	9 vs 6	[19]	Elevated	Enrolled
GSE72904	C57BL/6	28d	3 vs 3	Unpublished	Elevated	Enrolled
GSE52796	B6.129	28d	6 vs 9	[20]	Elevated	Enrolled
GSE68518	Not shown	28d	4 vs 6	[21]	Elevated	Enrolled
GSE56348	C57BL/6	28d	5 vs 5	[22]	Elevated	Enrolled

**Table 2:** GO items of the 16 shared DEGs with the same trends during the late stage of cardiac remodeling.

Term	Count	%	*P* value	Genes
GO:0071260~cellular response to mechanical stimulus	4	25	2.72*E* − 05	NPPB, COL1A1, ANKRD1, NPPA
GO:0071560~cellular response to transforming growth factor beta stimulus	3	18.75	9.92*E* − 04	POSTN, COL1A1, ANKRD1
GO:0035582~sequestering of BMP in extracellular matrix	2	12.5	3.09*E* − 03	NBL1, FBN1
GO:0071356~cellular response to tumor necrosis factor	3	18.75	3.18*E* − 03	POSTN, COL1A1, ANKRD1
GO:0030308~negative regulation of cell growth	3	18.75	4.09*E* − 03	NPPB, FLCN, NPPA
GO:0007168~receptor guanylyl cyclase signaling pathway	2	12.5	6.95*E* − 03	NPPB, NPPA
GO:0061049~cell growth involved in cardiac muscle cell development	2	12.5	8.49*E* − 03	NPPB, NPPA
GO:0001666~response to hypoxia	3	18.75	9.39*E* − 03	NPPB, POSTN, NPPA
GO:0003085~negative regulation of systemic arterial blood pressure	2	12.5	1.00*E* − 02	NPPB, NPPA
GO:0006182~cGMP biosynthetic process	2	12.5	1.31*E* − 02	NPPB, NPPA

## Data Availability

All data analyzed in this study were downloaded from the public database: GEO.

## Abbreviations

TAC: Transverse aortic constriction
AS: Aortic stenosis
DEG: Differential expression gene
PPI: Protein–protein interaction
GO: Gene Ontology
BP: Biological process
DAVID: The Database for Annotation, Visualization, and Integrated Discovery.

## Supplementary Materials

Supplementary Materials Table S1. BP items of the 74 DEGs with same trend in the early stage of cardiac remodeling. Table S2. Shared DEGs of the 7 datasets of late stage cardiac remodeling. Figure S1. Network diagram showed the DEG in late stage. Table S3. BP items of the DEGs from at least 6 datasets in the late stage of cardiac remodeling.
