# Supplementary material for: Transcriptome Analysis of Hypertrophic Heart Tissues from Murine Transverse Aortic Constriction and Human Aortic Stenosis Reveals Key Genes and Transcription Factors Involved in Cardiac Remodeling Induced by Mechanical Stress
Source: Dis Markers. 2019 Oct 27;2019:5058313. doi: 10.1155/2019/5058313 (PMC6854968; doi:10.1155/2019/5058313)
Supplement: Supplementary Materials — Table S1. BP items of the 74 DEGs with same trend in the early stage of cardiac remodeling. Table S2. Shared DEGs of the 7 datasets of late stage cardiac remodeling. Figure S1. Network diagram showed the DEG in late stage. Table S3. BP items of the DEGs from at least 6 datasets in the late stage of cardiac remodeling. [file 5058313.f1.docx]

Supplemental materials

Transcriptome analysis of hypertrophic heart tissues from murine transverse aortic constriction and human aortic stenosis induced by mechanical stress reveals key genes and transcription factors involved in cardiac remodeling

[Cell Biology and Toxicology](https://link.springer.com/journal/10565)

Peng Yu1,#, Baoli Zhang1,#, Ming Liu2, Ying Yu2, Ji Zhao1, Chunyu Zhang1, Yana Li, Xue Yang1, Hong Jiang1,*, Yunzeng Zou1, Junbo Ge1.

1 Shanghai Institute of Cardiovascular Diseases, Shanghai Clinical Bioinformatics Research Institute, Zhongshan Hospital, Shanghai Medical College of Fudan University

2 Department of General Practice, Zhongshan Hospital, Shanghai Medical College of Fudan University, Shanghai, China

#These authors contributed equally to this article

*Correspondence to: Hong JIANG, MD, PhD

E-mails: jiang.hong@zs-hospital.sh.cn

Address: Room 502, Building 5, Zhongshan Hospital, Fenglin Road 180, Xuhui District, Shanghai, China.

Tel./Fax: +86-21-64038038

Table S1. BP items of the 74 DEGs with same trend in the early stage of cardiac remodeling.

| Term | Count | % | *P* Value | Genes |
| --- | --- | --- | --- | --- |
| GO:0006631~fatty acid metabolic process | 7 | 9.59 | 3.114E-05 | SLC27A1, ECH1, UCP3, ACADS, MLYCD, DECR1, GHR |
| GO:0006629~lipid  metabolic process | 9 | 12.33 | 3.858E-04 | SLC27A1, ECH1, ACADS, MLYCD, ENPP2, CES1D, DECR1, PLA2G5, FDFT1 |
| GO:0008152~metabolic process | 9 | 12.33 | 4.087E-04 | BCKDHA, GCDH, SLC27A1, ACSS1, ECH1, ACADS, IVD, ENPP2, FDFT1 |
| GO:0033539~fatty acid beta-oxidation using acyl-CoA dehydrogenase | 3 | 4.11 | 2.421E-03 | GCDH, ACADS, IVD |
| GO:0055114~oxidation-reduction process | 9 | 12.33 | 4.484E-03 | BCKDHA, GCDH, SORD, FAM213A, ACADS, IVD, CREG1, DECR1, FDFT1 |
| GO:0050873~brown fat cell differentiation | 3 | 4.11 | 7.651E-03 | RGS2, SLC2A4, SELENBP1 |
| GO:0055088~lipid homeostasis | 3 | 4.11 | 1.099E-02 | GCDH, ACADS, IVD |
| GO:0006085~acetyl-CoA biosynthetic process | 2 | 2.74 | 1.921E-02 | ACSS1, MLYCD |
| GO:0060998~regulation of dendritic spine development | 2 | 2.74 | 2.301E-02 | FMR1, ARF6 |
| GO:0044829~positive regulation by host of viral genome replication | 2 | 2.74 | 2.679E-02 | STOM, PPIB |
| GO:0032868~response to insulin | 3 | 4.11 | 3.092E-02 | SLC27A1, EGR2, UCP3 |
| GO:0007049~cell cycle | 7 | 9.59 | 3.138E-02 | UHRF1, RGS2, CKS2, ARF6, CDC20, PMP22, MCM5 |
| GO:0051289~protein homotetramerization | 3 | 4.11 | 3.336E-02 | ACADS, DECR1, TK1 |
| GO:0006810~transport | 13 | 17.81 | 4.594E-02 | CREBRF, SLC27A1, FMR1, AQP4, ARF6, SELENBP1, CACNA1S, ABCC9, UCP3, SLC2A4, LASP1, PITPNC1, SEC61A1 |
| GO:0060397~JAK-STAT cascade involved in growth hormone signaling pathway | 2 | 2.74 | 4.919E-02 | SOCS3, GHR |

TableS2. Shared DEGs of the 7 datasets of late stage cardiac remodeling.

| Names | \| total \| \| --- \| | \| elements \| \| --- \| |
| --- | --- | --- | --- | --- |
| GSE12337, GSE2459, GSE48811, GSE52796, GSE56348, GSE68518, GSE72904 | 21 | Postn, Vim, Acta1, Fgl2, Fxyd5, Fbn1, Col8a1, Ankrd1, Col1a1, Mmp14, Nppb, Lifr, Mfap5, Nbl1, Dcn, Nppa, P3h4, Pfkp, Cpxm2, Flcn, Ces1d |
| GSE12337, GSE2459, GSE48811, GSE52796, GSE56348, GSE68518 | 1 | Vps29 |
| GSE12337, GSE2459, GSE48811, GSE52796, GSE68518, GSE72904 | 2 | Ptpn22, Auh |
| GSE12337, GSE2459, GSE48811, GSE56348, GSE68518, GSE72904 | 3 | Mgll, Cyp2b10, Nav1 |
| GSE2459, GSE48811, GSE52796, GSE56348, GSE68518, GSE72904 | 57 | AI464131, Prkcdbp, Crat, Nnt, Tgfb3, Ndufs3, Fbln2, Mgp, Aldob, Ube2e3, Npr3, Npc1, Kcnh2, E2f1, Fkbp4, Cyb561, Col3a1, Pcolce, Comt, Gab2, Adam9, Tcf4, Fhl1, Psme1, Adcy6, Emp1, Dsg2, Loxl1, Rbp1, Igfbp6, Lum, Hrc, Trim30a, Cd9, Crlf1, Fstl1, Etfb, Hbegf, Ghr, F2r, Cyr61, Ptger4, Cd34, Bgn, Entpd5, Slc4a3, Dio2, Col5a2, Apbb1, Serping1, Kcna1, Ctgf, Fbxw7, Eci1, Rtn2, Ndufv1, Stat5a |
| GSE12337, GSE48811, GSE52796, GSE56348, GSE68518, GSE72904 | 51 | Timp1, Heatr5b, Synj2, Slc39a6, Pcx, Dennd4a, Tbx15, Bcl2, Ednrb, Polr1a, Pip4k2a, Fmod, Slc25a1, Vcan, Plat, Kcnj5, Rarb, Parp1, St6galnac4, Rap1gap2, Gas2l3, Tmem201, Fam73b, Kcna2, Pam, Slc1a5, Cd93, Ppic, Clic5, Ubxn10, Tlr4, Cd44, Pex6, Nckap1l, Spred1, Sort1, Tnc, Neo1, Aqp8, Abi2, Atp2a2, Slc1a3, Capg, 4930402H24Rik, Cpt2, Gna12, Impa2, Sparc, Cobll1, Sntb2, Kcnd2 |
|  |  |  |

FigureS1. Network diagram showed the DEG in late stage.


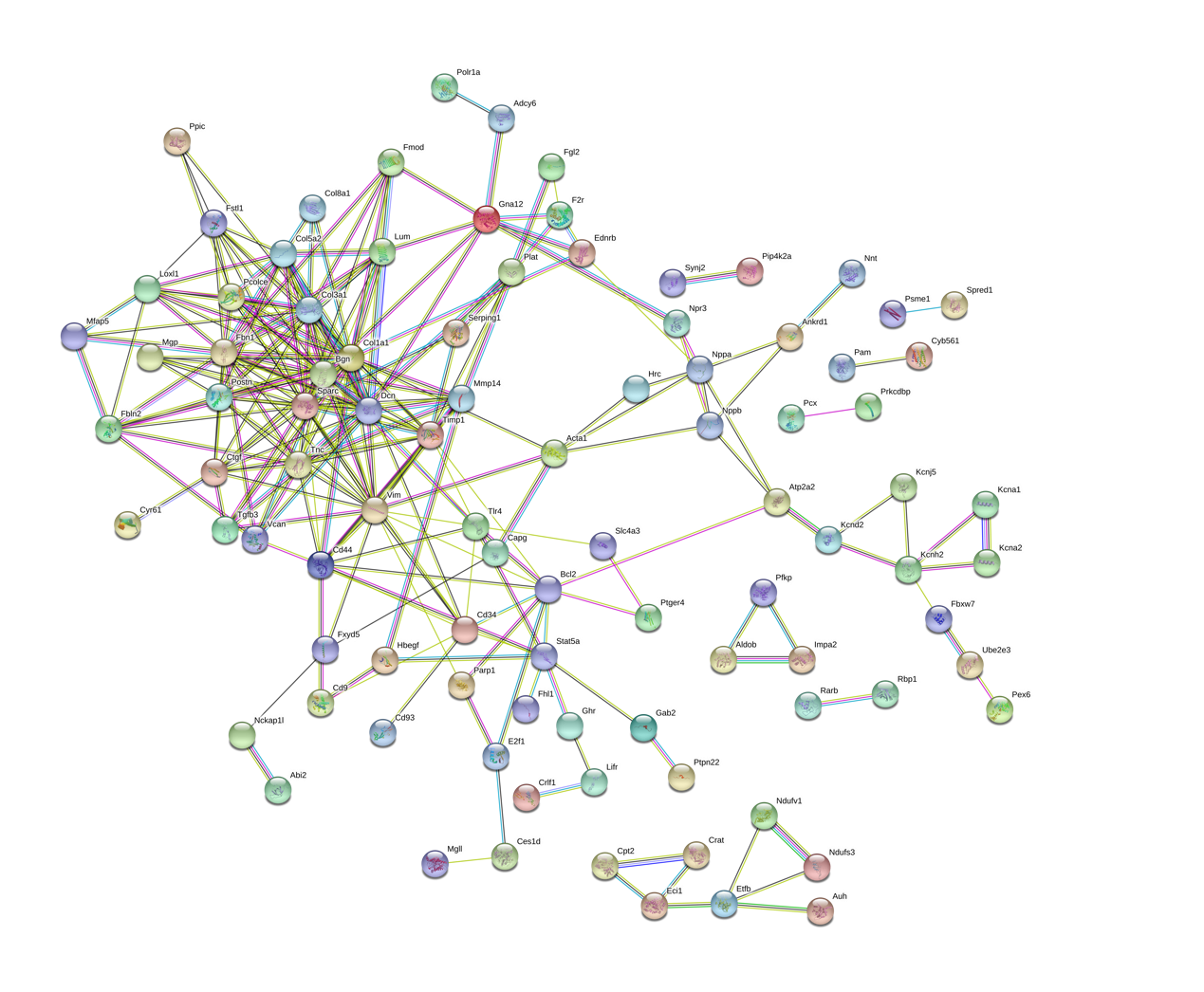


Table S3. BP items of the DEGs from at least 6 datasets in the late stage of cardiac remodeling .

| Term | Count | % | *p* Value | Genes |
| --- | --- | --- | --- | --- |
| GO:0043434~response to peptide hormone | 8 | 5.97 | 7.92E-07 | ATP2A2, CTGF, STAT5A, ALDOB, NPPB, COL1A1, SPARC, TIMP1 |
| GO:0032496~response to lipopolysaccharide | 10 | 7.46 | 1.06E-05 | EDNRB, PTGER4, PTPN22, NPPB, TLR4, SPARC, COMT, DCN, LOXL1, F2R |
| GO:0042060~wound healing | 7 | 5.22 | 5.24E-05 | TNC, COL3A1, TGFB3, COL1A1, SPARC, DCN, TIMP1 |
| GO:0001503~ossification | 7 | 5.22 | 5.90E-05 | CTGF, BCL2, SORT1, MGP, SPARC, MMP14, COL5A2 |
| GO:0009612~response to mechanical stimulus | 6 | 4.48 | 8.01E-05 | ACTA1, TNC, COL3A1, POSTN, DCN, MMP14 |
| GO:0035810~positive regulation of urine volume | 4 | 2.99 | 1.16E-04 | EDNRB, PTGER4, NPPB, NPR3 |
| GO:0072593~reactive oxygen species metabolic process | 5 | 3.73 | 1.42E-04 | NNT, CTGF, BCL2, NDUFS3, CYR61 |
| GO:0030199~collagen fibril organization | 5 | 3.73 | 1.58E-04 | FMOD, LUM, COL3A1, COL1A1, COL5A2 |
| GO:0007155~cell adhesion | 13 | 9.70 | 1.60E-04 | TNC, POSTN, NEO1, CD9, DSG2, CD93, CD44, CTGF, CD34, VCAN, COL8A1, CYR61, ADAM9 |
| GO:0050714~positive regulation of protein secretion | 5 | 3.73 | 1.74E-04 | PTGER4, TGFB3, ANKRD1, APBB1, ADAM9 |
| GO:0051930~regulation of sensory perception of pain | 5 | 3.73 | 1.92E-04 | EDNRB, MGLL, TLR4, COMT, F2R |
| GO:0071260~cellular response to mechanical stimulus | 6 | 4.48 | 2.21E-04 | PTGER4, NPPB, TLR4, COL1A1, ANKRD1, NPPA |
| GO:0032720~negative regulation of tumor necrosis factor production | 5 | 3.73 | 2.32E-04 | TRIM30A, PTGER4, CD34, PTPN22, TLR4 |
| GO:0030308~negative regulation of cell growth | 7 | 5.22 | 2.53E-04 | FHL1, BCL2, NPPB, NDUFS3, FLCN, APBB1, NPPA |
| GO:0043434~response to peptide hormone | 8 | 5.97 | 7.92E-07 | ATP2A2, CTGF, STAT5A, ALDOB, NPPB, COL1A1, SPARC, TIMP1 |
